# Interventions to reduce deaths in people living with HIV admitted to hospital in low- and middle-income countries: A systematic review

**DOI:** 10.1371/journal.pgph.0001557

**Published:** 2023-02-22

**Authors:** Rachael M. Burke, Hussein H. Twabi, Cheryl Johnston, Marriott Nliwasa, Ankur Gupta-Wright, Katherine Fielding, Nathan Ford, Peter MacPherson, Elizabeth L. Corbett

**Affiliations:** 1 Clinical Research Department, London School of Hygiene and Tropical Medicine, London, United Kingdom; 2 Malawi Liverpool Wellcome Clinical Research Programme, Blanytre, Malawi; 3 Helse Nord Tuberculosis Initiative, Kamuzu University of Health Science, Blantyre, Malawi; 4 Global HIV, Hepatitis, STI Programme, World Health Organisation, Geneva, Switzerland; 5 Division of Infection and Immunity, University College London, London, United Kingdom; 6 Department of Infectious Disease Epidemiology, London School of Hygiene and Tropical Medicine, London, United Kingdom; 7 School of Health and Wellbeing, University of Glasgow, Glasgow, United Kingdom; 3ie Dehli: International Initiative for Impact Evaluation Dehli, ZIMBABWE

## Abstract

People living with HIV (PLHIV) admitted to hospital have a high risk of death. We systematically appraised evidence for interventions to reduce mortality among hospitalised PLHIV in low- and middle-income countries (LMICs). Using a broad search strategy with terms for HIV, hospitals, and clinical trials, we searched for reports published between 1 Jan 2003 and 23 August 2021. Studies of interventions among adult HIV positive inpatients in LMICs were included if there was a comparator group and death was an outcome. We excluded studies restricted only to inpatients with a specific diagnosis (e.g. cryptococcal meningitis). Of 19,970 unique studies identified in search, ten were eligible for inclusion with 7,531 participants in total: nine randomised trials, and one before-after study. Three trials investigated systematic screening for tuberculosis; two showed survival benefit for urine TB screening vs. no urine screening, and one which compared Xpert MTB/RIF versus smear microscopy showed no difference in survival. One before-after study implemented 2007 WHO guidelines to improve management of smear negative tuberculosis in severely ill PLHIV, and showed survival benefit but with high risk of bias. Two trials evaluated complex interventions aimed at overcoming barriers to ART initiation in newly diagnosed PLHIV, one of which showed survival benefit and the other no difference. Two small trials evaluated early inpatient ART start, with no difference in survival. Two trials investigated protocol-driven fluid resuscitation for emergency-room attendees meeting case-definitions for sepsis, and showed increased mortality with use of a protocol for fluid administration. In conclusion, ten studies published since 2003 investigated interventions that aimed to reduce mortality in hospitalised adults with HIV, and weren’t restricted to people with a defined disease diagnosis. Inpatient trials of diagnostics, therapeutics or a package of interventions to reduce mortality should be a research priority.

**Trial registration: PROSPERO Number:** https://www.crd.york.ac.uk/prospero/display_record.php?ID=CRD42019150341.

## Introduction

Advanced HIV disease is a persistent public health challenge [1]. Expanded access to HIV testing and antiretroviral therapy (ART) has saved millions of lives globally, and probably averted many hospital admissions [2]. However, people living with HIV (PLHIV) continue to make up a disproportionate number of inpatient admissions in many high-HIV-burden countries, and of inpatients living with HIV many have advanced HIV disease (CD4 cells under 200 cells/mm^3^) [3–6]. Increasingly, inpatients admitted with advanced HIV disease have been previously diagnosed and started on ART, rather than being newly diagnosed with HIV, but have either stopped taking ART or have treatment failure [7–10], often related to virological resistance [3,11]. Under these circumstances, the causes and outcomes of hospital admissions may be relatively unchanged by the availability of ART in communities, even if hospitalisation rates and the proportion of admissions accounted for by PLHIV decline [4,12].

PLHIV who require admission to hospital are an important population who have a very high risk of death [13] and whose care needs are likely to be different from ambulatory PLHIV due to clinical acuity, high probability of opportunistic infections, and high risk of death. Overall in-hospital mortality risk for PLHIV was 20% in a systematic review [13]. For people who survive initial hospital admission, death within 12 months of discharge from hospital was 14.1% in another review [10]. In 2017 WHO released the first guidelines for management of advanced HIV [14] which were incorporated into consolidated ART guidelines in 2021 [15]. These guidelines acknowledged that the majority of available evidence informing interventions for managing patients with advanced HIV relates to ambulatory ART-naïve participants, and that more research is required to evaluate the optimal interventions for PLHIV with treatment failure and inpatient management [14].

To identify interventions with potential to reduce mortality in this group we systematically reviewed the existing literature relating to interventions aiming to reduce mortality in adult hospitalised PLHIV in low- and middle-income countries (LMICs). We aimed to review interventions broadly applicable to all or many hospitalised patients, rather than interventions offered to a sub-group where a definite aetiological diagnosis was already made.

## Methods

### Search strategy

The protocol and search strategy are available online at PROSPERO (CRD42019150341). We used a broad search strategy (S1 Appendix) to identify studies that recruited hospitalised HIV positive participants [13,16]. We searched MEDLINE, EMBASE OVID, and Cochrane Central databases and included papers published after 1 January 2003 as this was when ART became available in low-income countries [17]. We also hand-searched abstracts from Conference on Retroviruses and Opportunistic Infections (CROI) and International AIDS Society (IAS) between 2015 and 2021. The initial search was on 9^th^ October 2019, with an updated search on 23^rd^ August 2021.

### Eligibility criteria

Studies were eligible for inclusion if they reported results from a trial or study with a comparator arm (i.e. both randomised and non-randomised designs) that recruited inpatient adults (aged 15 years and older) living with HIV and where death was reported as an outcome (either inpatient mortality or measured over any specified time period following enrolment). We excluded studies solely in surgical or obstetric wards. This review was limited to adults, given the substantially different causes of paediatric HIV related hospital admissions [13].

We included studies with both inpatients and outpatients, both HIV positive and HIV-negative participants, and both adults and children, provided it was possible to disaggregate the data for inpatient adults living with HIV. We restricted studies to those conducted in LMICs as defined by the World Bank in 2019 [18]. Studies in sub-populations that required a specific aetiological diagnosis to have been made by clinicians in addition to HIV were excluded (for example, trials of participants with cryptococcal meningitis) as the aim here was to investigate broadly applicable interventions aimed at improving all-cause mortality. This was a change from original protocol which stated trials about interventions for some specific diagnosis would be included. This change was reflected in an amendment to the PROSPERO protocol on 20th March 2020.

### Screening and data extraction

Two reviewers (RMB and HT) screened titles and abstracts against inclusion criteria using Rayaan software [19]. For the October 2019 search both reviewers assessed all title-abstracts and full text reviews independently in duplicate. For the August 2021 extension, 10% of title-abstracts and all full text articles were reviewed in duplicate. Differences between reviewers were resolved by discussion and consensus including a third reviewer (PM). Where studies included participants who met our review inclusion criteria but results were not disaggregated (most commonly, when both inpatients and outpatients were included), we contacted the authors by email to ask for disaggregated results. We extracted data from eligible studies on study year, location, trial design, intervention, comparator, median CD4 cell counts, proportion of participants on ART, proportion with tuberculosis, mortality outcome definition and deaths by trial arm.

### Risk of bias assessment

We used the Cochrane Risk of Bias (ROB) 2 tool to assess study quality of individually randomised studies [20]. For cluster randomised trials we used Cochrane Risk of Bias (2016) [21]. For non-randomised studies we used the Cochrane ROBINS-I tool (Risk Of Bias In Non-randomised Studies of Interventions) [22].

### Synthesis of results

Results are presented in narrative format, with summary measures as reported in the relevant papers or by authors’ communication. We report adjusted estimates if these were performed by authors. Where no summary measure was reported for the population and outcome of interest, we calculated unadjusted absolute risk differences from grouped data. No meta-analysis was performed as study interventions and populations were too diverse. We grouped trials by the population recruited–whether all PLHIV in hospital, newly diagnosed PLHIV or PLHIV with additional set of symptoms or other criteria (for example, PLHIV with tuberculosis [TB] symptoms).

## Results

**Fig 1:**
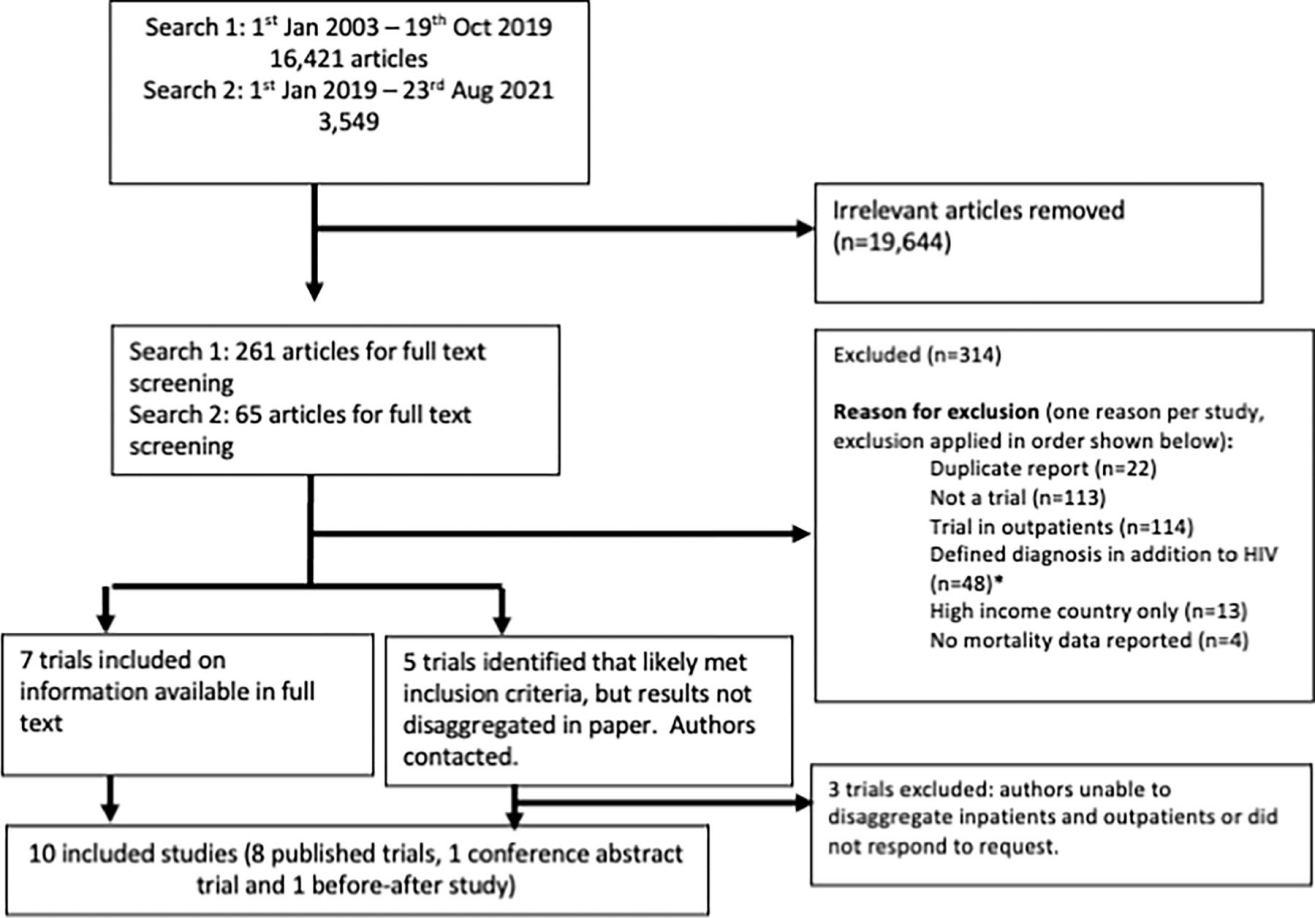
PRISMA diagram. *Defined diagnoses were cryptococcal meningitis (29 studies), TB (including TB meningitis, TB pericarditis and TB IRIS, 10 studies in total), bacterial meningitis (2 studies), Pneumocystis jirovecci pneumonia (2 studies), and bacterial pneumonia, toxoplasmic encephalitis, visceral leishmaniasis, Kaposi-sarcoma IRIS, and progressive multifocal leukoencephalopathy (1 study each).

After removing duplicates, we identified 16,421 studies from our initial search, from which 262 articles were selected for full text screening. In an updated search in August 2021, we identified 3,549 further articles for title-abstract screening and 64 for full text review. Nine published studies and one conference abstract were included in our qualitative synthesis (Fig 1).

Table 1 summarises the characteristics of included studies. Three studies were conducted in Zambia, three in South Africa and one study each in Malawi, Uganda, Tanzania, Zimbabwe, Brazil, China and Mexico (two of the ten studies were multi-country). There were 7531 participants in total (range 58 to 2574 participants per study). Median CD4 count ranged from 40 cells/mm^3^ in a Zambian trial that recruited patients with suspected severe sepsis during 2012, to 227 cells/mm^3^ in a trial that recruited all PLHIV admitted to general medical wards in Malawi and South Africa during 2015–2017. For six studies not restricted to newly diagnosed HIV, the proportion of participants on ART ranged from 16% (in South Africa, recruitment between 2008–2009) to 72% (in Malawi and South Africa, recruitment 2015–2017). Overall, 23% of participants had died by the end of study follow up: follow up duration ranged from time of discharge from hospital to 12 months from enrolment.

**Table 1:** Characteristics of included studies.

Study	Design	Year(s) of trial	County or countries	Setting	Population (whole trial)	Number participants (whole trial) *	Number participants who were adult inpatients with HIV	Median CD4 counts (cells/mm^3^) #	% on ART at enrolment	Intervention	Comparator	Primary outcome	Mortality outcome details (if not primary outcome)
**Studies in all inpatients with HIV**													
Gupta-Wright et al. (STAMP) (2018) [23]	Individually randomised. Double blind.	2015–2017	Malawi and South Africa	2 hospitals (1 DGH / 1 referral)	Inpatient adults living with HIV (regardless of symptoms)	2574	2574	227	72%	Systematic screening for TB using urine LAM and sputum Xpert MTB/rif	Systematic screening for TB using sputum Xpert MTB/rif alone.	All-cause mortality at 56 days.	NA
**Studies in newly diagnosed inpatients with HIV**													
Wanyenze et al. (2013) [24]	Individually randomised. Not blinded.	2008–2011	Uganda	1 referral hospital.	Inpatient and outpatient adults newly diagnosed with HIV.	965	342	Not stated	NA	“Enhanced linkage to care”. This involved counselling, supported disclosure, accompaniment to ART clinic.	Usual care. This involved giving an appointment card for ART clinic on discharge.	Receipt of HIV care. Defined as attending ART clinic.	All cause mortality at 90 days and at 12 months.
Wu et. al. (2017) [25]	Cluster randomised, hospital as unit of randomisation. Not blinded.	2015	China	12 hospitals (all DGHs)	Inpatient and outpatient adults newly diagnosed with HIV.	487	338	Not stated	NA	“One4all programme”. Involves using rapid tests for HIV confirmation, CD4 count and HIV viral load in parallel.	Usual care. Usual care involved step-wise at reference labs with long turn around time.	“Completeness of testing” within 30 days. Defined as having HIV antibodies, CD4 and viral load available and post-test counselling.	All cause mortality at 90 days and at 12 months.
Boniatti et. al. (2020) [26]	Individually randomised. Not blinded.	2012–2015	Brazil	Intensive care unit at one hospital.	Adults admitted to ICU newly diagnosed with HIV with CD<350 or AIDS defining illness.	115	115	37 in intervention group, 49 in control group.	NA	Starting ART within 5 days of enrolment in trial.	Starting ART after discharge from ICU.	All-cause mortality prior to discharge from hospital.	In ICU morality and 6-month mortality also reported.
Peralta-Prado et. al. (2021) [27]	Individually randomised. Not blinded.	Not stated	Mexico	1 hospital	Adults admitted with an “opportunistic disease” (details no specified)	58	58	56	NA	“Immediate ART” (median of 2 days from enrolment to ART in this group)	“Conventional delayed ART” (median of 11 days to ART in this group)	Survival at 360 days	NA
**Studies in inpatients with HIV and TB symptoms**													
Holtz et al. (2011) [29]	Before and after study. Not randomised or blinded.	2008–2009	South Africa	3 hospitals (1 DGH, 1 private, 1 hybrid private/DGH)	Adult inpatients living with HIV who had “suspected pulmonary tuberculosis” and WHO danger signs.	525	525	122 pre-intervention group, 74 in control group.	16%	Management “according to the 2007 WHO recommended algorithm for diagnosis and treatment of smear-negative pulmonary tuberculosis”.	Management “according to standard practice”.	Continued stay in hospital at 7 days and survival at 56 days after admission.	All cause mortality at 56 days.
Peter et al. (LAM-RCT) (2016) [28]	Individually randomised. Not blinded.	2013–2014	South Africa, Zambia, Tanzania, Zimbabwe	10 hospitals (mix of DGH and referral)	Inpatient adults living with HIV with any WHO TB symptom.	2528	2528	84	48%	Systematic urine LAM testing plus routine diagnostic TB tests.	Routine diagnostic TB tests only. (These varied between sites).	All-cause mortality at 18 weeks.	NA
**Studies in inpatients with HIV and signs and symptoms consistent with sepsis**													
Andrews et al (2014) [30	Individually randomised. Not blinded.	2012	Zambia	1 referral hospital emergency department.	Adults with suspected infection + SIRs + organ dysfunction.	109	88	70 in control group, 40 in intervention group.	38%	“Early goal directed therapy”. This was up to 4 litres IV fluid and dopamine and/or blood transfusion in selected patients.	Usual care.	In-hospital all-cause mortality.	NA
Andrews et al (2017) [31]	Individually randomised. Not blinded.	2012–2013	Zambia	1 referral hospital emergency department.	Adults with suspected infection + SIRS + hypotension. Participants with hypoxia or tachypnoea were excluded.	209	187	65 in control group. 72 in intervention group.	57%	“An early resuscitation protocol for sepsis”. This was up to 4 litres IV fluid and dopamine and/or blood transfusion in selected patients.	Usual care	In-hospital all-cause mortality.	NA
**Studies in inpatients with HIV mechanically ventilated in intensive care**													
Calligaro et al. (2015) [32]	Individually randomised. Not blinded.	2010–2013	South Africa	Intensive care units at 4 hospitals (mix DGH and referral).	Mechanically ventilated adults with either HIV (regardless of symptoms) or HIV negative with TB symptoms.	341	115	122 in control group, 190 in intervention group.	28%	Local Xpert + culture respiratory secretions.	Smear microscopy plus culture of respiratory secretions preformed at a reference lab.	Proportion of culture-positive participants started on TB treatment 48 hours after enrolment.	All cause mortality in intensive care, in hospital, at 28 days, and at 90 days.

DGH = District General Hospital; SIRS Systemic Inflammatory Response Syndrome; LAM Lipoarabinomannan.

* Several trials included inpatients and outpatients, or inpatients with and without HIV.

# Only if reported for inpatients only (some trials which included both inpatients and outpatients didn’t report this data).

Table 2 summarises the interventions assessed and their impact on mortality. Risk of bias assessments are detailed in S1 Appendix.

**Table 2:** Effect of interventions to reduce mortality among adult PLHIV inpatients.

Study	Intervention	Comparison	Outcomes for adult PLHIV admitted to hospital, disaggregated from main trial population where necessary.				LTFU (%)	Overall risk of bias ^#^	Source of presented data and effect estimate.
			Time-period over which mortality was ascertained	Mortality in comparison / control arm	Mortality in intervention arm	Measure of association between intervention and mortality outcome and 95% confidence interval *			
**All PLHIV**									
Gupta-Wright et al. (STAMP) (2018) [23]	Systematic urine LAM and sputum Xpert MTB/rif screening for TB.	Systematic screening for TB with sputum MTB/rif alone.	56 days	272/1287 (21%)	235 / 1287 (18%)	aRD -2.8%, 95% CI -5.8% - 0.3%). Adjusted for site only.	29 (1.1%)	Low	Published study.
**Newly diagnosed PLHIV**									
Wanyenze et al. (2013) [24]	Enhanced linkage to care (see for description)	Usual care	3 months 1 year	22 / 168 (13%) 36/168 (21%)	30 / 174 (17%) 47 / 174 (27%)	RD +4% [-3% to + 12%] RD + 6% [-3% to + 15%]	87 (9%)^	Some concerns	Disaggregated mortality outcome from author. Effect estimate calculated from grouped data.
Wu et. al. (2017) [25]	One4All programme (see text for description)	Usual care	1 year	98 / 185 (53%)	54 / 153 (35%)	RD -18% [-28% to -7%]	34 (6.9%)^	Low	Disaggregated mortality outcomes from published paper. Effect estimate calculated from grouped data, ignoring clustering (may be falsely narrow).
Bonaiatti et. al. (2020)	Early ART start (within 5 days)	ART start after ICU discharge.	In hospital	38 / 57 (69%)	37 / 58 (64%)	RD +3% [-15% to +20%]	5 (4.3%)	Some concerns	Published study, effect estimate calculated.
Peralto-Prado et. al. (2021)	Early ART	Deferred ART	360 days	2/ 28 (7%)	4 / 30 (13%)	RD -6% [-22%—+9%]	Not stated	Some concerns	Published study, effect estimate calculated.
**PLHIV with signs and symptoms of TB or with presumptive TB.**									
Holtz et al. (2011) [29]	“Implementation of WHO 2007 TB treatment guidelines”	Usual care prior to institutional implementation of WHO 2007 TB treatment guidelines.	In hospital At 8 weeks	30 / 338 (9%) 108 / 338 (32%)	12 / 187 (6%) 31 / 187 (17%)	RR 0.57 (0.328–1.14) aRR 0.46 (0.30–0.70) Adjusted for hospital and baseline CD4 count.	No participants LTFU	High	Published study.
Peter et al. (LAM-RCT) (2016)	Systematic urine LAM testing plus routine diagnostic TB tests.	Routine diagnostic TB tests only.	56 days	317 / 1271 (25%)	261 / 1257 (21%)	aRR 0.83 (0.73–0.96) Adjusted for country of recruitment.	117 (4.6%)	Some concerns	Published study.
**PLHIV with signs of symptoms consistent with sepsis**									
Andrews et al (2014) [30]	Early Goal Directed Therapy for sepsis with hypotension (see text)	Usual care	In-hospital	29 / 46 (63%)	29 / 42 (69%)	RR 1.10 (0.81–1.48)	6 (5.5%)^	Low	Disaggregated mortality outcome and effect estimate from published paper.
Andrews et al (2017) [31]	Early Goal Directed Therapy for sepsis with hypotension (see text)	Usual care	In hospital	29 / 93 (31%)	46 / 94 (49%)	RR 1.57 (1.09–2.26)	15 (7.2%)^	Low	Disaggregated mortality outcome and effect estimate from published paper.
**PLHIV who are mechanically ventilated**									
Calligaro et al. (2015) [32]	Systematic TB screening using local Xpert MTB/rif testing.	Systematic TB screening using central reference lab culture.	28 days 90 days	12 / 30 (40%) 12 / 30 (40%)	13 / 37 (35%) 16 / 37 (43%)	RD -5% (-28% to 18%) RD 3.2% (-20% to 27%)	11 (3.2%)^	Low	Disaggregated mortality data from author, effect estimate calculated.

* Measure of association is according the primary outcome for paper or main mortality outcome reported (where mortality is not a primary trial outcome). Were there is no measure of association in the published paper (usually because PLHIV or inpatients were a subgroup) we calculate an estimated risk difference and 95% confidence interval from grouped data, ignoring clustering if relevant.

# Risk of bias assessed using Cochrane ROB 2019 for randomised trials and Cochrane ROBINS-i for non-randomised studies.

^ Applies to the whole trial population and not just inpatient adults with HIV (unable to disaggregate LTFU).

**Abbreviations**: a = adjusted, OR = Odds Ratio, RR = Risk Ratio, HR = Hazard Ratio, RD = Risk difference, LTFU = Lost to follow up.

### Interventions relevant to all people living with HIV admitted to hospital

Only one study, the STAMP trial (2018), recruited all PLHIV admitted to adult medical wards regardless of presenting signs or symptoms. Gupta-Wright and colleagues randomly allocated HIV positive inpatient participants to receive systematic screening for tuberculosis with either urine lipoarabinomannan (LAM) plus urine Xpert MTB/Rif (Xpert) plus sputum Xpert, or with sputum Xpert alone [23]. Overall, the difference in mortality at 56 days post enrolment between the two arms was consistent with survival benefit in the group with urine TB testing (adjusted risk difference for death [aRD] -2.8%, 95% CI -5.8% to 0.3%, not statistically significant). In three pre-specified sub-groups there was a survival advantage in the group screened with urine diagnostics. These were in participants with CD4 cell counts <100 cells/mm^3^ (aRD -7.1%, -13.7% to -0.4%), participants with haemoglobin < 8g/dL (aRD –9.0%, –16.6% to –1.3%), and participants with tuberculosis in differential diagnosis (aRD –5.7% –10.9% to –0.5%). Tuberculosis was microbiologically confirmed in 16% (210/1287) of participants randomised to the urine diagnostic arm, based on combined results from the admission urine and sputum Xpert screen plus other usual care diagnostics on clinician request. This was a blinded trial, with low risk of bias.

### Interventions relevant to newly diagnosed PLHIV hospital inpatients

Four studies evaluated interventions relevant to people newly diagnosed with HIV at the time of admission.

Wanyenze and colleagues (2013) randomised participants who tested HIV positive at a tertiary referral hospital in Uganda to standard vs. enhanced linkage to care [24]. Enhanced linkage to care involved counselling to reduce barriers to linkage to care, optional assisted disclosure to people who could provide social support, in-person introduction to clinic location and clinic staff and a reminder by phone call or home visit about their scheduled ART clinic visit, which for inpatients was following discharge. Approximately one-third of patients were inpatients at the time of randomisation. For all participants (outpatients and inpatients) there was no difference between standard linkage to care and enhanced linked to care (aHR 0.97, 95% CI 0.70–1.36). The authors provided data for participants recruited as inpatients or from emergency departments at our request. Compared to standard linkage to care, enhanced linkage to care did not alter mortality at one year, although the confidence interval was wide (unadjusted risk difference [RD] +6%, 95% CI -3% to +15%). There were some concerns of bias due to completeness of follow up (9% of participants were lost to follow up).

Wu and colleagues (2017) evaluated an intervention package that removed laboratory test barriers to timely ART initiation [25]. Twelve hospitals in China were randomised to either usual care or to implementation of the “One4All” intervention. All people (inpatients and outpatients) newly diagnosed with HIV on the basis of a single screening lateral flow HIV test in each hospital were recruited to the study. The intervention involved parallel rapid HIV antibody confirmatory testing, point of care CD4 count and viral load measurement at baseline. Usual care involved sequential testing for these three tests, with long turnaround times and requiring multiple clinic visits for blood tests, as was standard of care at the time. The hospital policies were that results of these tests were required before ART could be commenced. In the intervention arm required tests were complete in a median of 12 days from enrolment, compared with 58 days in usual care arm, meaning that the intervention reduced the time before ART was started. By 12 months 35% (54/153) of inpatient participants in the One4All group had died, compared to 53% (98/185) in the control group; this corresponded to an unadjusted RD for mortality of -18% (95% CI, -28% to -7%). This confidence interval was calculated from grouped data and ignores effects of clustering. This trial had a low risk of bias.

Boniatti and colleagues (2020) investigated early (within five days from enrolment) compared to late (after ICU discharge) ART start among ART-naïve HIV positive participants admitted to ICU in Brazil with low CD4 count or a AIDS-defining illness [26]. Timing of ART initiation made no difference to death by six months, RD +3% [-15% to +20%] for early ART compared to late ART. Forty-three percent (51/118) of people had diagnosed tuberculosis, including microbiologically confirmed and clinically diagnosed TB. There were some concerns of bias due to loss to follow up. In a conference abstract, Peralta-Prado and colleagues report results of a trial which randomly allocated 58 adult PLHIV with opportunistic infections not on ART to immediate vs. delayed ART in hospital (median 2 days to ART initiation in immediate arm and 11 days in deferred arm) [27]. There was no difference in survival at one year from enrolment.

### Interventions relevant to people based on defined symptoms and signs

We identified five studies that evaluated interventions targeted towards patients with specific constellations of clinical symptoms and signs.

Two studies were conducted among PLHIV with signs or symptoms of TB. In the LAM-RCT study, Peter and colleagues (2016) randomised participants with any WHO TB symptom (cough, fever, weight loss or night sweats) in ten hospitals in four countries (Zambia, Zimbabwe, South Africa and Tanzania) to either urine LAM screening or routine non-LAM diagnostic tests for TB [28]. Urine LAM screening reduced all-cause mortality at eight weeks (RD -4%, 95% CI -7 to -1%). TB was microbiologically confirmed in 26% (664/2528) of all participants by TB culture or Xpert (corresponding to 29% (664/2333) of all participants who had at least one Xpert or culture sample taken). There were some concerns of bias due to the unblinded design.

In a non-randomised before-and-after study conducted in 2008–2009, Holtz and colleagues (2011) investigated the effect of implementing the WHO 2007 smear-negative and extrapulmonary TB diagnosis and treatment guidelines for PLHIV at three hospitals in South Africa [29]. Hospitalised PLHIV were recruited if they were seriously ill (one or more WHO “danger signs”), had TB symptoms or a chest X-ray consistent with TB and negative sputum smear microscopy (the authors refer to this as meeting definition for smear negative TB, although in the pre-intervention part of the study less than half of participants actually received a TB diagnosis). In the pre-intervention group (August 2008 to February 2009), usual care varied and was not guided by any protocol; in the intervention period (March to December 2009) clinicians were trained and asked to manage patients according to the WHO 2007 algorithm which recommended empirical initiation of TB treatment. The percentage of participants initiating TB treatment was 46% (157/338) in the pre-intervention cohort and 100% (187/187) in the intervention cohort. Mortality was much lower in the post-intervention cohort compared to pre-intervention, with an adjusted hazard ratio (aHR) for death up to 56 days from enrolment of 0.46 (95% CI: 0.30–0.70) after adjustment for baseline CD4 count and hospital. This was an unblinded before-and-after study with serious risk of bias.

Two studies recruited patients with signs and symptoms consistent with sepsis and hypotension; both were RCTs conducted in one hospital in Zambia that evaluated the use of early goal-directed therapy in the emergency department. In both trials, adults attending the hospital emergency department were eligible regardless of HIV status, but the majority were HIV positive. In their first trial Andrews and colleagues (2014) recruited participants with signs and symptoms consistent with sepsis and organ dysfunction between February and July 2012 [30]. In the second trial (Andrews et al, 2017) [31] recruitment was restricted to people attending the emergency department between October and December 2013 with hypotension, but without hypoxia or tachypnoea. The intervention protocol mandated up to four litres intravenous fluid, and vasopressors or blood transfusion in participants with refractory hypotension and anaemia respectively. In both trials, a higher proportion of participants in the intervention groups died compared to those in the control groups: risk ratio (RR) 1.05, 95%CI 0.79–1.41 for the first trial (not statistically significant), and RR 1.46, 95% CI 1.04–2.05 for second trial. The authors note that limited access to intensive care unit level monitoring or mechanical ventilation for patients may have contributed to harm from aggressive fluid resuscitation. Among all participants in both trials (including a small proportion HIV-negative), 27% (81/297) had microbiologically confirmed TB. These trials both had some concerns of bias due to the unblinded design, although this would have likely had the effect of bias towards the null.

One trial (Calligaro et al, 2015) investigated a TB screening intervention comprising Xpert MTB/Rif testing compared to smear microscopy done in a centralised laboratory among ventilated patients in ICU in South Africa, regardless of symptoms or reason for ICU admission [32]. The prevalence of microbiologially confirmed TB was high (15%, 13/86 participants overall across both trial arms). Use of Xpert compared to smear was not associated with a difference in mortality between groups at 90 days (RD +3%, 95% CI -20% to 27%), and this trial was terminated early due to Xpert becoming standard of care in South Africa. This trial was at a low risk of bias.

## Discussion

People living with HIV who are unwell enough to require hospital admission likely have healthcare needs that are substantially different to people attending outpatient services, due to clinical acuity, extremely high probability of opportunistic infections and high risk of poor outcomes [10,13]. Whilst the numbers of people living with HIV being admitted to hospital are likely decreasing due to community availability of ART, and newer ART regimens [12], people in hospital contribute disproportionately to AIDS deaths. For example, about one quarter of all AIDS related deaths in Blantyre, Malawi in 2018 occurred in a government hospital [12]. There is no evidence of improvement in outcomes for PLHIV admitted to hospital over time [10,12,13]. While the WHO 2017 guidelines for advanced HIV disease and 2021 consolidated guidelines [14,15] apply to both inpatients and outpatients, most of the evidence considered for those guidelines was from outpatient studies, and there are currently no differentiated recommendations to reflect the particular needs of inpatients. We identified ten publications from 2003 (nine randomised trials and one before-and-after study) that investigated interventions aimed broadly at reducing mortality among PLHIV admitted to hospital in LMICs, although several of these were interventions that are superseded by more recent ART guidelines. Four consistent findings in studies in review were: high risk of death, with early mortality ranging from 20% (at 56 days, all PLHIV admitted to medical wards in South Africa and Malawi) [23] to 70% (by six months, PLHIV in intensive care in Brazil) [26]; a high prevalence of microbiologically confirmed TB; high prevalence of signs of critical illness; and low CD4 counts even in studies where many participants are on ART. The finding of low CD4 counts persisted in more recent studies even though many participants in more recent studies [23,28,31] reported already knowing their HIV status and taking ART (Table 2). Interventions were aimed at intensified TB diagnosis or treatment (four studies), reducing perceived or actual barriers to initiation of ART (two studies), rapid inpatient ART start (Two studies), and fluid-based management of sepsis (two studies).

TB is a major cause of death in PLHIV admitted to hospital, as shown in previous reviews of hospitalised HIV cohorts [13,33] and autopsy series [34]. Consistent with this: *M*. *tuberculosis* was the single most common blood culture pathogen in the sepsis trials in Zambia (isolated from 35% of positive blood cultures); microbiologically confirmed tuberculosis was diagnosed in 16% of urine-screening-arm STAMP trial participants (unselected PLHIV admitted to medical wards in Malawi and South Africa) [23]; 27% of urine screening arm LAM-RCT participants (PLHIV inpatients with TB symptoms in Tanzania, South Africa, Zambia and Zimbabwe); and 15% of PLHIV ventilated in Intensive Treatment Unit (ITU) in South Africa (regardless of reason for ITU admission). Four of the ten studies investigated TB-related interventions [23,28,29,32]. Two multi-country randomised trials [23,28] investigated TB urine diagnostic interventions and reported substantially increased proportions of inpatients treated for TB as well as modest mortality reductions. These results led to a strong recommendation from WHO in 2019 [35] that urine LAM testing should be used to test all inpatients with CD4 <200 cells/mm^3^, signs and symptoms of TB or who are seriously unwell [35–37]. Although we did not identify any trials investigating empirical TB treatment specifically for inpatients, one non-randomised study in South Africa [29] reported large survival gains from implementing the 2007 WHO algorithm for critically ill PLHIV with TB symptoms; which in effect substantially increased the number of people starting TB treatment without clinical confirmation of TB. However, this study was before widespread availability of sputum Xpert and urine LAM testing for TB. Both TB prevalence and risk of death from TB vary considerably by level of health service for PLHIV [38], thus these results support the need for randomised trials investigating inpatient empirical TB treatment, despite the lack of benefit shown for outpatients [39–41]. Such studies could be combined with investigation of more intensive TB regimens, for instance high dose rifampicin, and would ideally use pragmatic eligibility criteria aiming to recruit large numbers of inpatients, as for LAM-RCT and STAMP, rather than aiming for highly pre-screened and/or immunosuppressed participants that can cause critical recruitment problems [42] and applicability concerns.

A high proportion of participants in all trials had clinical signs of critical illness. Nearly half of all emergency department attendees in the Zambian trials met the case definition for sepsis [30,31] The definitions of sepsis used for these studies are similar to WHO danger signs used to identify need for hospitalisation. In the STAMP trial, 21% of all participants (all PLHIV in medical wards) had one or more WHO ‘danger signs’ at the time of recruitment [23].

Of the six studies that included people regardless of ART status, there was a pronounced increase from the earlier to the later trials in the proportion of participants already being on ART, reflecting global efforts to scale-up diagnosis and treatment as part of UNAIDS Fast-track HIV elimination strategy. Most trials reported median CD4 count and proportion of participants on ART, but without disaggregating CD4 counts by ART status. Median CD4 counts were low, ranging from 40 cells/mm^3^ for a 2012 study with ART at admission in 38% of participants [32] to 227 cells/mm^3^ for a 2015–2017 study with ART at admission in 72%. [23] Low median CD4 count despite high ART coverage suggests a high prevalence of underlying ART treatment failure, confirmed for patients admitted to the Malawi site of the STAMP trial [11]. Given the high mortality for admitted PLHIV, we recommend that investigation and management of potential ART treatment failure should be considered as a matter of urgency for inpatients, ideally informed by trials investigating health outcomes from diagnostic interventions providing early data on viral load, genotyping, and investigating optimal timing of ART regimen change following inpatient admission. Such trials could inform current WHO HIV treatment failure guidelines that are mainly based on data from ambulant outpatients.

Two of the included trials [24,25] addressed real or perceived barriers to starting ART; one showed a mortality benefit and one didn’t. These were all before 2016 “treat all” recommendations and some of the barriers that were being overcome (such as availability of CD4 counts to determine ART eligibility) are no longer standard of care [15]. Two small studies of very early vs. slightly delayed ART showed no difference in mortality (with wide confidence intervals).

Limitations to our present study include trials for which our population of interest (hospitalised PLHIV) represented only a proportion of the entire trial population, leaving trials underpowered for inpatient mortality. In two papers, authors were unable to disaggregate the inpatients and outpatients thus these were not included [43]. We did not include trials that were restricted to people in hospital who already had a specific diagnosed opportunistic infection (for example cryptococcal meningitis). We had initially intended to include interventions for people with certain diagnosed opportunistic infections but after starting the search we amended the inclusion criteria (reflected in changes at PROSPERO registration) for three reasons. First, we were initially including several small drug trials of varying quality and were thereby duplicating work of other reviews (for example, cryptococcal meningitis, where clear guidelines based on a systematic review about appropriate management already exist). Second, because, microbiologically or pharmacologically, treatment for diagnosed opportunistic infections isn’t likely to substantially vary between inpatients and outpatients and inpatients aren’t necessarily a distinct population–although they have a higher risk of death. Finally, interventions delivered to a small subset of inpatients are unlikely to have large public health benefits, and our focus was on interventions or a package of interventions that could be recommended generally for hospitalised adults to broadly reduce risk of death.

In summary, we found relatively few trials to inform recommendations for interventions or a package of interventions to offer as part of a package of care to adults living with HIV admitted to hospital. There were two studies of urine TB diagnostics which showed mortality reductions in people with TB symptoms, but no studies of other diagnostic interventions. Understanding the likely impact of combined diagnostic interventions such as urine LAM, cryptococcal antigen tests, rapid CD4 count and viral load measurements would help to guide investment in inpatient management. This could be similar to studies such as REMSTART [44] and REALITY [45] which informed the 2017 WHO recommended package of care for advanced HIV [14] while the package could be delivered to both inpatients and outpatients with advanced HIV, the evidence is from trials in outpatients. There is a need for evidence to optimise the timely diagnosis and management of potential ART treatment failure in inpatients—people who are unwell enough to be admitted to hospital and who are currently taking ART might have worse outcomes than those not on ART, so this is a priority [46]. Whilst a trial in Uganda over a decade ago didn’t show a difference in survival for people offered a linkage to care package (many of whom weren’t eligible for ART at the time) vs. no linkage to care, more evidence in the “treat all” era about the best strategies to start, restart or change ART, or otherwise address ART treatment failure, and effectively link people from hospitals to primary care should be another priority. Given the high prevalence of TB among inpatient PLHIV and sub-optimal sensitivity of urine LAM tests, trials of empirical anti-infective treatment of critically ill PLHIV would be helpful, for instance empirical TB treatment with or without additional antibiotics for severe bacterial infection.

### Conclusion

Overall mortality was 23% (range 15% to 66%) for participants in this heterogeneous group of inpatient studies from different continents, with different recruitment criteria and different follow up durations. TB screening using urine-based diagnostics reduced mortality, and there was a suggestion of benefit from empirical TB treatment among inpatients that should be investigated further despite the lack of benefit shown for outpatients. Aggressive fluid administration for people who are critically ill should generally be avoided especially where intensive care facilities are not readily available. PLHIV who require hospitalisation should be managed with urgency, with results from trials specific to inpatients ideally used to guide optimal management and improve patient outcomes.

## Supporting information

S1 Appendix Search strategy and ROB Medline search strategy, and risk of bias assessments. (DOCX)

S2 Appendix PRISMA checklist. (DOCX)

## References

[1] Calmy A, Ford N, Meintjes G. The Persistent Challenge of Advanced HIV Disease and AIDS in the Era of Antiretroviral Therapy. Clin Infect Dis. 2018;66: S103–SS105. doi: 10.1093/cid/cix1138 29514231 PMC5850411

[2] Granich R, Gupta S, Hersh B, Williams B, Montaner J, Young B, et al. Trends in AIDS Deaths, New Infections and ART Coverage in the Top 30 Countries with the Highest AIDS Mortality Burden; 1990–2013. PLoS ONE. 2015;10: e0131353. doi: 10.1371/journal.pone.0131353 26147987 PMC4493077

[3] Ousley J, Niyibizi AA, Wanjala S, Vandenbulcke A, Kirubi B, Omwoyo W, et al. High Proportions of Patients With Advanced HIV Are Antiretroviral Therapy Experienced: Hospitalization Outcomes From 2 Sub-Saharan African Sites. Clin Infect Dis. 2018;66: S126–S131. doi: 10.1093/cid/ciy103 29514239 PMC5850537

[4] Meintjes G, Kerkhoff AD, Burton R, Schutz C, Boulle A, Van Wyk G, et al. HIV-Related Medical Admissions to a South African District Hospital Remain Frequent Despite Effective Antiretroviral Therapy Scale-Up. Medicine (Baltimore). 2015;94. doi: 10.1097/MD.0000000000002269 26683950 PMC5058922

[5] Matoga MM, Rosenberg NE, Stanley CC, LaCourse S, Munthali CK, Nsona DP, et al. Inpatient mortality rates during an era of increased access to HIV testing and ART: A prospective observational study in Lilongwe, Malawi. PLoS One. 2018;13: e0191944. doi: 10.1371/journal.pone.0191944 29415015 PMC5802850

[6] Barak T, Neo DT, Tapela N, Mophuthegi P, Zash R, Kalenga K, et al. HIV-associated morbidity and mortality in a setting of high ART coverage: prospective surveillance results from a district hospital in Botswana. J Int AIDS Soc. 2019;22: e25428. doi: 10.1002/jia2.25428 31850683 PMC6918506

[7] Coelho LE, Ribeiro SR, Japiassu AM, Moreira RI, Lara PC, Veloso VG, et al. Thirty-day Readmission Rates in an HIV-infected Cohort From Rio de Janeiro, Brazil. J Acquir Immune Defic Syndr. 2017;75: e90–e98. doi: 10.1097/QAI.0000000000001352 28291051 PMC5484736

[8] Cichowitz C, Pellegrino R, Motlhaoleng K, Martinson NA, Variava E, Hoffmann CJ. Hospitalization and post-discharge care in South Africa: A critical event in the continuum of care. PLoS One. 2018;13: e0208429. doi: 10.1371/journal.pone.0208429 30543667 PMC6292592

[9] Haachambwa L, Kandiwo N, Zulu PM, Rutagwera D, Geng E, Holmes CB, et al. Care Continuum and Postdischarge Outcomes Among HIV-Infected Adults Admitted to the Hospital in Zambia. Open Forum Infect Dis. 2019;6: ofz336. doi: 10.1093/ofid/ofz336 31660330 PMC6778319

[10] Ford N, Patten G, Rangaraj A, Davies M-A, Meintjes G, Ellman T. Outcomes of people living with HIV after hospital discharge: a systematic review and meta-analysis. The Lancet HIV. 2022;9: e150–e159. doi: 10.1016/S2352-3018(21)00329-5 35245507 PMC8905089

[11] Gupta-Wright A, Fielding K, van Oosterhout JJ, Alufandika M, Grint DJ, Chimbayo E, et al. Virological failure, HIV-1 drug resistance, and early mortality in adults admitted to hospital in Malawi: an observational cohort study. Lancet HIV. 2020;7: e620–e628. doi: 10.1016/S2352-3018(20)30172-7 32890497 PMC7487765

[12] Burke RM, Henrion MYR, Mallewa J, Masamba L, Kalua T, Khundi M, et al. Incidence of HIV-positive admission and inpatient mortality in Malawi [2012–2019]: a population cohort study. AIDS. 2021. doi: 10.1097/QAD.0000000000003006 34172671 PMC7611991

[13] Ford N, Shubber Z, Meintjes G, Grinsztejn B, Eholie S, Mills EJ, et al. Causes of hospital admission among people living with HIV worldwide: a systematic review and meta-analysis. Lancet HIV. 2015;2: e438–444. doi: 10.1016/S2352-3018(15)00137-X 26423651

[14] World Health Organization. Guidelines for managing advanced HIV disease and rapid initiation of antiretroviral therapy. 2017. Available: http://www.ncbi.nlm.nih.gov/books/NBK475977/. 29341560

[15] Organization WH. Guidelines: updated recommendations on HIV prevention, infant diagnosis, antiretroviral initiation and monitoring. World Health Organization; 2021. Available: https://apps.who.int/iris/handle/10665/340190. 33822559

[16] 6.4.11.2 Search filters for identifying randomized trials in EMBASE and 6.3.2.2 What is in The Cochrane Central Register of Controlled Trials (CENTRAL) from EMBASE? Cochrane Handbook for Systematic Reviews of Interventions. [cited 10 Sep 2019]. Available: https://handbook-5-1.cochrane.org/.

[17] Treating 3 million by 2005: making it happen: the WHO strategy. Geneva: World Health Organization: Joint United Nations Programme on HIV/AIDS; 2003.

[18] World Bank Country and Lending Groups–World Bank Data Help Desk. [cited 16 Jun 2020]. Available: https://datahelpdesk.worldbank.org/knowledgebase/articles/906519-world-bank-country-and-lending-groups.

[19] Ouzzani Mourad, Hammady Hossam, Fedorowicz Zbys, and Elmagarmid Ahmed. Rayyan—a web and mobile app for systematic reviews. Systematic Reviews (2016) 5:210, doi: 10.1186/s13643-016-0384-4 [cited 6 Sep 2019]. Available: https://rayyan.qcri.org/welcome. 27919275 PMC5139140

[20] Sterne JAC, Savović J, Page MJ, Elbers RG, Blencowe NS, Boutron I, et al. RoB 2: a revised tool for assessing risk of bias in randomised trials. BMJ. 2019;366: l4898. doi: 10.1136/bmj.l4898 31462531

[21] Revised Cochrane risk of bias tool for randomized trials (RoB 2.0): additional considerations for cluster-randomized trials. / Eldridge, Sandra; Campbell, Marion; Campbell, Michael; Drahota-Towns, Amy; Giraudeau, Bruno; Higgins, Julian; Reeves, Barney; Siegfried, Nandi. 2016. Available: https://sites.google.com/site/riskofbiastool/welcome/rob-2-0-tool/archive-rob-2-0-cluster-randomized-trials-2016?authuser=0.

[22] Sterne JA, Hernán MA, Reeves BC, Savović J, Berkman ND, Viswanathan M, et al. ROBINS-I: a tool for assessing risk of bias in non-randomised studies of interventions. BMJ. 2016;355. doi: 10.1136/bmj.i4919 27733354 PMC5062054

[23] Gupta-Wright A, Corbett EL, Oosterhout JJ van, Wilson D, Grint D, Alufandika-Moyo M, et al. Rapid urine-based screening for tuberculosis in HIV-positive patients admitted to hospital in Africa (STAMP): a pragmatic, multicentre, parallel-group, double-blind, randomised controlled trial. The Lancet. 2018;392: 292–301. doi: 10.1016/S0140-6736(18)31267-4 30032978 PMC6078909

[24] Wanyenze RK, Kamya MR, Fatch R, Mayanja-Kizza H, Baveewo S, Szekeres G, et al. Abbreviated HIV counselling and testing and enhanced referral to care in Uganda: a factorial randomised controlled trial. The Lancet Global Health. 2013;1: e137–e145. doi: 10.1016/S2214-109X(13)70067-6 25104262 PMC4129546

[25] Wu Z, Tang Z, Mao Y, Veldhuisen PV, Ling W, Liu D, et al. Testing and linkage to HIV care in China: a cluster-randomised trial. The Lancet HIV. 2017;4: e555–e565. doi: 10.1016/S2352-3018(17)30131-5 28867267 PMC6639122

[26] Boniatti MM, Pellegrini JAS, Marques LS, John JF, Marin LG, Maito LRDM, et al. Early antiretroviral therapy for HIV-infected patients admitted to an intensive care unit (EARTH-ICU): A randomized clinical trial. PLOS ONE. 2020;15: e0239452. doi: 10.1371/journal.pone.0239452 32956419 PMC7505451

[27] Peralta-Prado AB. Immediate versus delayed antiretroviral treatment in hospitalized persons with AIDS-defining opportunistic disease: a randomized clinical trial. IAS 2021. Available: https://theprogramme.ias2021.org/Abstract/Abstract/531.

[28] Peter JG, Zijenah LS, Chanda D, Clowes P, Lesosky M, Gina P, et al. Effect on mortality of point-of-care, urine-based lipoarabinomannan testing to guide tuberculosis treatment initiation in HIV-positive hospital inpatients: a pragmatic, parallel-group, multicountry, open-label, randomised controlled trial. Lancet. 2016;387: 1187–1197. doi: 10.1016/S0140-6736(15)01092-2 26970721

[29] Holtz TH, Kabera G, Mthiyane T, Zingoni T, Nadesan S, Ross D, et al. Use of a WHO-recommended algorithm to reduce mortality in seriously ill patients with HIV infection and smear-negative pulmonary tuberculosis in South Africa: an observational cohort study. Lancet Infect Dis. 2011;11: 533–540. doi: 10.1016/S1473-3099(11)70057-3 21514234

[30] Andrews B, Muchemwa L, Kelly P, Lakhi S, Heimburger D, Bernard G. Simplified Severe Sepsis Protocol: A Randomized Controlled Trial of Modified Early Goal–Directed Therapy in Zambia*. Critical Care Medicine. 2014;42: 2315–2324. doi: 10.1097/CCM.0000000000000541 25072757 PMC4199893

[31] Andrews B, Semler MW, Muchemwa L, Kelly P, Lakhi S, Heimburger DC, et al. Effect of an Early Resuscitation Protocol on In-hospital Mortality Among Adults With Sepsis and Hypotension. JAMA. 2017;318: 1233–1240. doi: 10.1001/jama.2017.10913 28973227 PMC5710318

[32] Calligaro GL, Theron G, Khalfey H, Peter J, Meldau R, Matinyenya B, et al. Burden of tuberculosis in intensive care units in Cape Town, South Africa, and assessment of the accuracy and effect on patient outcomes of the Xpert MTB/RIF test on tracheal aspirate samples for diagnosis of pulmonary tuberculosis: a prospective burden of disease study with a nested randomised controlled trial. The Lancet Respiratory Medicine. 2015;3: 621–630. doi: 10.1016/S2213-2600(15)00198-8 26208996

[33] Ford N, Matteelli A, Shubber Z, Hermans S, Meintjes G, Grinsztejn B, et al. TB as a cause of hospitalization and in-hospital mortality among people living with HIV worldwide: a systematic review and meta-analysis. J Int AIDS Soc. 2016;19. doi: 10.7448/IAS.19.1.20714 26765347 PMC4712323

[34] Gupta RK, Lucas SB, Fielding KL, Lawn SD. Prevalence of tuberculosis in post-mortem studies of HIV-infected adults and children in resource-limited settings: a systematic review and meta-analysis. AIDS. 2015;29: 1987–2002. doi: 10.1097/QAD.0000000000000802 26266773 PMC4568896

[35] Lateral flow urine lipoarabinomannan assay (LF-LAM) for the diagnosis of active tuberculosis in people living with HIV, 2019 Update. [cited 8 Dec 2021]. Available: https://www.who.int/publications-detail-redirect/9789241550604.

[36] Bjerrum S, Schiller I, Dendukuri N, Kohli M, Nathavitharana RR, Zwerling AA, et al. Lateral flow urine lipoarabinomannan assay for detecting active tuberculosis in people living with HIV. Cochrane Database of Systematic Reviews. 2019 [cited 13 Dec 2021]. doi: 10.1002/14651858.CD011420.pub3 31633805 PMC6802713

[37] Nathavitharana RR, Lederer P, Chaplin M, Bjerrum S, Steingart KR, Shah M. Impact of diagnostic strategies for tuberculosis using lateral flow urine lipoarabinomannan assay in people living with HIV. Cochrane Database of Systematic Reviews. 2021 [cited 22 Sep 2022]. doi: 10.1002/14651858.CD014641 34416013 PMC8407503

[38] Nliwasa M, MacPherson P, Gupta-Wright A, Mwapasa M, Horton K, Odland JØ, et al. High HIV and active tuberculosis prevalence and increased mortality risk in adults with symptoms of TB: a systematic review and meta-analyses. J Int AIDS Soc. 2018;21: e25162. doi: 10.1002/jia2.25162 30063287 PMC6067081

[39] Grant AD, Charalambous S, Tlali M, Karat AS, Dorman SE, Hoffmann CJ, et al. Algorithm-guided empirical tuberculosis treatment for people with advanced HIV (TB Fast Track): an open-label, cluster-randomised trial. Lancet HIV. 2020;7: e27–e37. doi: 10.1016/S2352-3018(19)30266-8 31727580 PMC7617063

[40] Blanc F-X, Badje AD, Bonnet M, Gabillard D, Messou E, Muzoora C, et al. Systematic or Test-Guided Treatment for Tuberculosis in HIV-Infected Adults. N Engl J Med. 2020;382: 2397–2410. doi: 10.1056/NEJMoa1910708 32558469

[41] Hosseinipour MC, Bisson GP, Miyahara S, Sun X, Moses A, Riviere C, et al. Empirical tuberculosis therapy versus isoniazid in adult outpatients with advanced HIV initiating antiretroviral therapy (REMEMBER): a multicountry open-label randomised controlled trial. Lancet. 2016;387: 1198–1209. doi: 10.1016/S0140-6736(16)00546-8 27025337 PMC4931281

[42] Manabe YC, Worodria W, van Leth F, Mayanja-Kizza H, Traore AN, Ferro J, et al. Prevention of Early Mortality by Presumptive Tuberculosis Therapy Study: An Open Label, Randomized Controlled Trial. Am J Trop Med Hyg. 2016;95: 1265–1271. doi: 10.4269/ajtmh.16-0239 27928077 PMC5154437

[43] Sierra-Madero JG, Ellenberg SS, Rassool MS, Tierney A, Belaunzaran-Zamudio PF, Lopez-Martinez A, et al. Effect of the CCR5 antagonist maraviroc on the occurrence of immune reconstitution inflammatory syndrome in HIV (CADIRIS): a double-blind, randomised, placebo-controlled trial. Lancet HIV. 2014;1: e60–7. doi: 10.1016/S2352-3018(14)70027-X 26366430 PMC4563882

[44] Mfinanga S, Chanda D, Kivuyo SL, Guinness L, Bottomley C, Simms V, et al. Cryptococcal meningitis screening and community-based early adherence support in people with advanced HIV infection starting antiretroviral therapy in Tanzania and Zambia: an open-label, randomised controlled trial. Lancet. 2015;385: 2173–2182. doi: 10.1016/S0140-6736(15)60164-7 25765698

[45] Hakim J, Musiime V, Szubert AJ, Mallewa J, Siika A, Agutu C, et al. Enhanced Prophylaxis plus Antiretroviral Therapy for Advanced HIV Infection in Africa. New England Journal of Medicine. 2017;377: 233–245. doi: 10.1056/NEJMoa1615822 28723333 PMC5603269

[46] Gupta-Wright A, Corbett EL, Wilson D, van Oosterhout JJ, Dheda K, Huerga H, et al. Risk score for predicting mortality including urine lipoarabinomannan detection in hospital inpatients with HIV-associated tuberculosis in sub-Saharan Africa: Derivation and external validation cohort study. Hatherill M, editor. PLoS Med. 2019;16: e1002776. doi: 10.1371/journal.pmed.1002776 30951533 PMC6450614

