# Supplementary material for: Interventions to reduce deaths in people living with HIV admitted to hospital in low- and middle-income countries: A systematic review
Source: PLOS Glob Public Health. 2023 Feb 22;3(2):e0001557. doi: 10.1371/journal.pgph.0001557 (PMC10022356; doi:10.1371/journal.pgph.0001557)
Supplement: S1 Appendix — (DOCX) [file pgph.0001557.s001.docx]

**S1 Appendix: Search Strategy and Risk of Bias Assessment**

**Medline search strategy**

| 1 | Terms related to HIV | 1. exp HIV/ 2. exp HIV Infections/ 3. (HIV or human immunodeficiency virus or HIV-1 or HIV-2 or AIDS or Acquired Immunodeficiency or acquired immun* deficiency).mp. 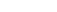 4. 4. 1 or 2 or 3 |
| --- | --- | --- |
| 2 | Terms related to inpatients (a) | 1. Inpatients/ 2. hospital*.mp. 3. patient*.mp. 4. inpatient*.mp 5. (admission* or readmission*).mp. 6. (admit* or readmit).mp. 7. exp Hospitalization/ 8. discharge*.mp 9. 5or6or7or8or9or10or11or12 |
| 3 | Filter for clinical trials (b) | 1. Randomi?ed controlled trial.pt. 2. controlled clinical trial.pt. 3. randomi?ed.ab. 4. placebo.ab. 5. clinical trials as topic.sh. 6. randomly.ab. 7. trial.ti. 8. 14or15or16or17or18or19or20 9. exp animals/ not humans.sh. 10. 21 not 22 |
| 4 | 1 and 2 and 3 and 4 | 1. 4 and 13 and 23 |
| 5 | Date of publication filter | 1. limit 24 to yr="2003 -Current" |

**Risk of Bias Assessment**

We assessed risk of bias (i.e. that the intervention truly caused the observed difference in mortality) using Cochrane ROB 2 ^1^ for individually randomised trials assessed on basis of assignment to intervention. Risk of bias assessments across domains is show in figure below, and signalling questions are listed underneath.
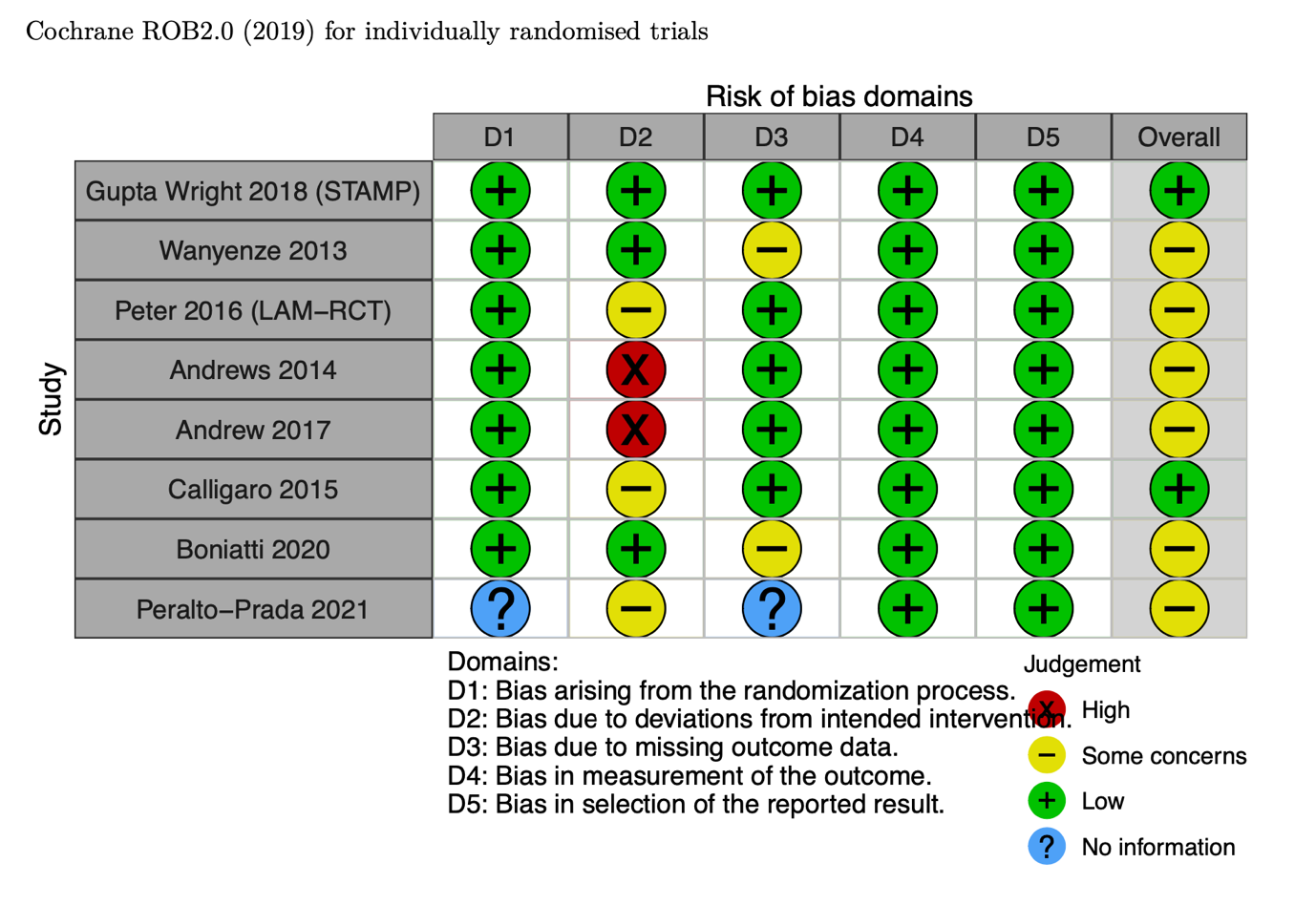


**Domain 1: Bias arising from the randomisation process**

- 1. Was the allocation sequence random?
  2. Was the allocation sequence concealed until participants were enrolled and assigned to interventions?
  3. Did baseline differences between intervention groups suggest a problem with the randomization process?

**Domain 2**: **Bias due to deviations from intended interventions**

2.1. Were participants aware of their assigned intervention during the trial?

2.2. Were carers and people delivering the interventions aware of participants' assigned intervention during the trial?

2.3. If Y/PY/NI to 2.1 or 2.2: Were there deviations from the intended intervention that arose because of the trial context?

2.4 If Y/PY to 2.3: Were these deviations likely to have affected the outcome?

2.5. If Y/PY/NI to 2.4: Were these deviations from intended intervention balanced between groups?

2.6 Was an appropriate analysis used to estimate the effect of assignment to intervention?

2.7 If N/PN/NI to 2.6: Was there potential for a substantial impact (on the result) of the failure to analyse participants in the group to which they were randomized?

**Domain 3: Bias due to missing outcome data**

3.1 Were data for this outcome available for all, or nearly all, participants randomized?

3.2 If N/PN/NI to 3.1: Is there evidence that the result was not biased by missing outcome data?

3.3 If N/PN to 3.2: Could missingness in the outcome depend on its true value?

3.4 If Y/PY/NI to 3.3: Is it likely that missingness in the outcome depended on its true value?

**Domain 4: Bias in measurement of the outcome**

4.1 Was the method of measuring the outcome inappropriate?

4.2 Could measurement or ascertainment of the outcome have differed between intervention groups?

4.3 If N/PN/NI to 4.1 and 4.2: Were outcome assessors aware of the intervention received by study participants?

4.4 If Y/PY/NI to 4.3: Could assessment of the outcome have been influenced by knowledge of intervention received?

4.5 If Y/PY/NI to 4.4: Is it likely that assessment of the outcome was influenced by knowledge of intervention received?

**Domain 5: Bias in selection of the reported result**

5.1 Were the data that produced this result analysed in accordance with a pre-specified analysis plan that was finalized before unblinded outcome data were available for analysis?

Is the numerical result being assessed likely to have been selected, on the basis of the results, from...

5.2. ... multiple eligible outcome measurements (e.g. scales, definitions, time points) within the outcome domain?

5.3 ... multiple eligible analyses of the data?

For the cluster randomised trial we used Cochrane ROB 2.0 (2016) for cluster randomised trials.^2^


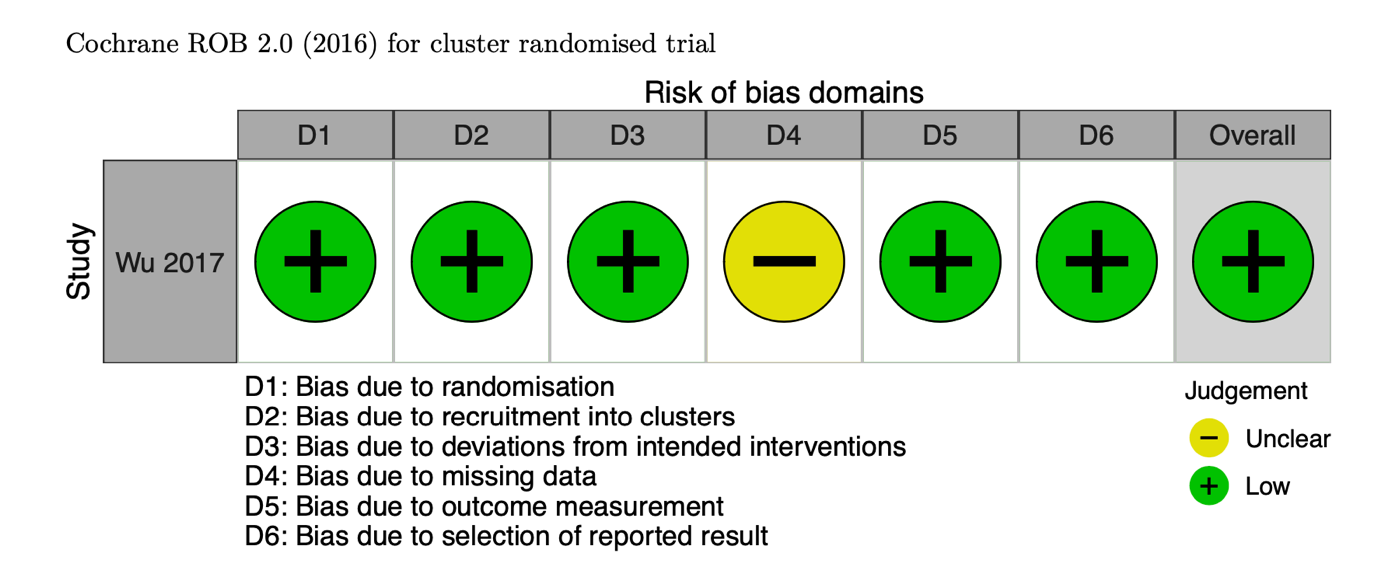


**Domain 1A: Bias arising from the randomisation process**

- 1. Was the allocation sequence random?
  2. Was the allocation sequence concealed until participants were enrolled and assigned to interventions?
  3. Did baseline differences between intervention groups suggest a problem withthe randomization process?

**Domain 1B: Bias arising from the timing of identification and recruitment of individual participants in relation to timing of randomization**

1b.1 Were all the individual participants identified before randomization of clusters (and if the trial specifically recruited patients were they all recruited before randomization of clusters)?

1b.2 If N/PN/NI to 1b.1: Is it likely that selection of individual participants was affected by knowledge of the intervention?

1b.3 Were there baseline imbalances that suggest differential identification or recruitment of individual participants between arms?

**Domain 2**: **Bias due to deviations from intended interventions**

2.1a Were participants aware that they were in a trial?

2.1b If Y/PY/NI to 2.1a:Were participants aware of their assigned intervention during the trial?

2.2. Were carers and people delivering the interventions aware of participants' assigned intervention during the trial?

2.3. If Y/PY/NI to 2.1 or 2.2: Were there deviations from the intended intervention beyond what would be expected in usual practice?

2.4. If Y/PY to 2.3: Were these deviations from intended intervention unbalanced between groups and likely to have affected the outcome?

2.5a Were any clusters analysed in a group different from the one to which they were assigned?

2.5b Were any participants analysed in a group different from the one to which their original cluster was randomized?

2.5. If Y/PY/NI to 2.4: Were these deviations from intended intervention balanced between groups?

2.6 Was an appropriate analysis used to estimate the effect of assignment to intervention?

2.7 If N/PN/NI to 2.6: Was there potential for a substantial impact (on the result) of the failure to analyse participants in the group to which they were randomized?

**Domain 3: Bias due to missing outcome data**

3.1a Were outcome data available for all, or nearly all, clusters randomized?

3.1b Were outcome data available for all, or nearly all, participants within clusters?

3.2 If N/PN/NI to 3.1a or 3.1b: Are the proportions of missing outcome data and reasons for missing outcome data similar across intervention groups?

3.3 If N/PN/NI to 3.1a or 3.1b: Is there evidence that results were robust to the presence of missing outcome data?

**Domain 4: Bias in measurement of the outcome**

4.1a Were outcome assessors aware that a trial was taking place?

4.1b If Y/PY/NI to 4.1: Were outcome assessors aware of the intervention received by study participants?

4.2 If Y/PY/NI to 4.1: Was the assessment of the outcome likely to be influenced by knowledge of intervention received?

**Domain 5: Bias in selection of the reported result**

Are the reported outcome data likely to have been selected, on the basis of the results, from...

5.1. ... multiple outcome measurements (e.g. scales, definitions, time points) within the outcome

domain?

5.2 ... multiple analyses of the data?

For the before-after study (Holtz et al 2011) we used Cochrane ROBINS-I tool.^3^ For ROBINS-I we compared to a target trial of clinician-guided TB treatment initiation with no specific guidelines vs. a protocol-driven approach where all people were recommended to start TB treatment, in a population of adult PLHIV inpatients with TB symptoms or a chest Xray suggestive of TB, and WHO danger signs.


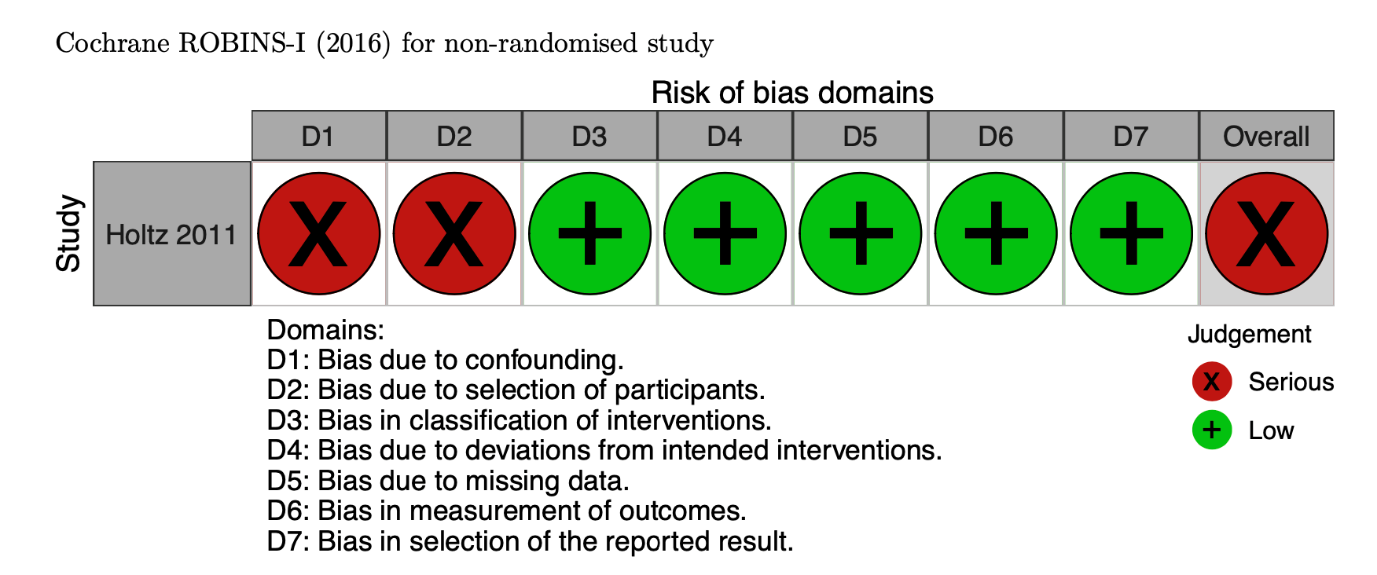


**Domain 1: Bias due to confounding**

1.1 Is there potential for confounding of the effect of intervention in this study?

If N/PN to 1.1: the study can be considered to be at low risk of bias due to confounding and no further signalling questions need be considered

If Y/PY to 1.1: determine whether there is a need to assess time-varying confounding:

1.2. Was the analysis based on splitting participants’ follow up time according to intervention received? If N/PN, answer questions relating to baseline confounding (1.4 to 1.6) If Y/PY, go to question 1.3.

1.3. Were intervention discontinuations or switches likely to be related to factors that are prognostic for the outcome? If N/PN, answer questions relating to baseline confounding (1.4 to 1.6) If Y/PY, answer questions relating to both baseline and time-varying confounding (1.7 and 1.8)

Questions relating to baseline confounding only

1.4. Did the authors use an appropriate analysis method that controlled for all the important confounding domains?

1.5. If Y/PY to 1.4: Were confounding domains that were controlled for measured validly and reliably by the variables available in this study?

1.6. Did the authors control for any post- intervention variables that could have been affected by the intervention?

Questions relating to baseline and time-varying confounding

1.7. Did the authors use an appropriate analysis method that controlled for all the important confounding domains and for time-varying confounding?

1.8. If Y/PY to 1.7: Were confounding domains that were controlled for measured validly and reliably by the variables available in this study?

**Bias in selection of participants into the study**

2.1. Was selection of participants into the study (or into the analysis) based on participant characteristics observed after the start of intervention? If N/PN to 2.1: go to 2.4

2.2. If Y/PY to 2.1: Were the post-intervention variables that influenced selection likely to be associated with intervention?

2.3 If Y/PY to 2.2: Were the post-intervention variables that influenced selection likely to be influenced by the outcome or a cause of the outcome?

2.4. Do start of follow-up and start of intervention coincide for most participants?

2.5. If Y/PY to 2.2 and 2.3, or N/PN to 2.4: Were adjustment techniques used that are likely to correct for the presence of selection biases?

**Domain 3: Bias in classification of interventions**

3.1 Were intervention groups clearly defined?

3.2 Was the information used to define intervention groups recorded at the start of the intervention?

3.3 Could classification of intervention status have been affected by knowledge of the outcome or risk of the outcome?

**Domain 4: Bias due to deviations from intended interventions**

4.1. Were there deviations from the intended intervention beyond what would be expected in usual practice?

4.2. If Y/PY to 4.1: Were these deviations from intended intervention unbalanced between groups and likely to have affected the outcome?

**Domain 5: Bias due to missing data**

5.1 Were outcome data available for all, or nearly all, participants?

5.2 Were participants excluded due to missing data on intervention status?

5.3 Were participants excluded due to missing data on other variables needed for the analysis?

5.4 If PN/N to 5.1, or Y/PY to 5.2 or 5.3: Are the proportion of participants and reasons for missing data similar across interventions?

5.5 If PN/N to 5.1, or Y/PY to 5.2 or 5.3: Is there evidence that results were robust to the presence of missing data?

**Domain 6: Bias in measurement of outcomes**

6.1 Could the outcome measure have been influenced by knowledge of the intervention received?

6.2 Were outcome assessors aware of the intervention received by study participants?

6.3 Were the methods of outcome assessment comparable across intervention groups?

6.4 Were any systematic errors in measurement of the outcome related to intervention received?

**Domain 7: Bias in selection of the reported result**

Is the reported effect estimate likely to be selected, on the basis of the results, from...

7.1. ... multiple outcome measurements within the outcome domain?

7.2 ... multiple analyses of the intervention-outcome relationship?

7.3 ... different subgroups?

**References:**

1. RoB 2: A revised Cochrane risk-of-bias tool for randomized trials | Cochrane Bias. https://methods.cochrane.org/bias/resources/rob-2-revised-cochrane-risk-bias-tool-randomized-trials.

2. Revised Cochrane risk of bias tool for randomized trials (RoB 2.0) : additional considerations for cluster-randomized trials. / Eldridge, Sandra; Campbell, Marion; Campbell, Michael; Drahota-Towns, Amy; Giraudeau, Bruno; Higgins, Julian; Reeves, Barney; Siegfried, Nandi. 2016.

3. Sterne, J. A. *et al.* ROBINS-I: a tool for assessing risk of bias in non-randomised studies of interventions. *BMJ* **355**, (2016).
